# Next-generation sequencing using microfluidic PCR enrichment for molecular autopsy

**DOI:** 10.1186/s12872-019-1154-8

**Published:** 2019-07-23

**Authors:** Hariharan Raju, James S. Ware, Jonathan R. Skinner, Paula L. Hedley, Gavin Arno, Donald R. Love, Christian van der Werf, Jacob Tfelt-Hansen, Bo Gregers Winkel, Marta C. Cohen, Xinzhong Li, Shibu John, Sanjay Sharma, Steve Jeffery, Arthur A. M. Wilde, Michael Christiansen, Mary N. Sheppard, Elijah R. Behr

**Affiliations:** 1 Cardiovascular Sciences Research Centre, Molecular and Clinical Sciences Research Institute, St George’s University of London, London, SW17 0RE UK; 2 Health Cardiology, Faculty of Medicine & Health Sciences, Macquarie University, Suite 203, 2 Technology Place, Sydney, NSW 2109 Australia; 3 NIHR Royal Brompton Cardiovascular Biomedical Research Unit, Royal Brompton Hospital, London, UK; 4 Greenlane Paediatric and Congenital Cardiac Services, Starship Childrens Hospital, Auckland, New Zealand; 5 Department for Congenital Disorders, Statens Serum Institut, Copenhagen, Denmark; 6 Diagnostic Genetics, Auckland City Hospital, Auckland, New Zealand; 7 Amsterdam UMC, Heart Centre, and Department of Clinical and Experimental Cardiology, Amsterdam Cardiovascular Sciences, University of Amsterdam, Amsterdam, Netherlands; 8 Department of Cardiology, Copenhagen University Hospital, Rigshospitalet, Denmark; 9 Department of Forensic Medicine, University of Copenhagen, København, Denmark; 10 Histopathology Department, Sheffield Children’s Hospital, Sheffield, UK; 11 School of Science, Engineering and Design, Teesside University, Middlesbrough, UK; 12 ERN-GUARD Heart (European Union), Brussels, Belgium; 13 Department of Biomedical Sciences, University of Copenhagen, Copenhagen, Denmark

**Keywords:** Molecular autopsy, Sudden arrhythmic death syndrome, Sudden unexplained death, Inherited cardiac conditions, Next generation sequencing

## Abstract

**Background:**

We aimed to determine the mutation yield and clinical applicability of “molecular autopsy” following sudden arrhythmic death syndrome (SADS) by validating and utilizing low-cost high-throughput technologies: Fluidigm Access Array PCR-enrichment with Illumina HiSeq 2000 next generation sequencing (NGS).

**Methods:**

We validated and optimized the NGS platform with a subset of 46 patients by comparison with Sanger sequencing of coding exons of major arrhythmia risk-genes (*KCNQ1*, *KCNH2*, *SCN5A*, *KCNE1*, *KCNE2*, *RYR2*). A combined large multi-ethnic international SADS cohort was sequenced utilizing the NGS platform to determine overall molecular yield; rare variants identified by NGS were subsequently reconfirmed by Sanger sequencing.

**Results:**

The NGS platform demonstrated 100% sensitivity for pathogenic variants as well as 87.20% sensitivity and 99.99% specificity for all substitutions (optimization subset, *n* = 46). The positive predictive value (PPV) for NGS for rare substitutions was 16.0% (27 confirmed rare variants of 169 positive NGS calls in 151 additional cases). The overall molecular yield in 197 multi-ethnic SADS cases (mean age 22.6 ± 14.4 years, 68% male) was 5.1% (95% confidence interval 2.0–8.1%), representing 10 cases carrying pathogenic or likely pathogenic risk-mutations.

**Conclusions:**

Molecular autopsy with Fluidigm Access Array and Illumina HiSeq NGS utilizing a selected panel of LQTS/BrS and CPVT risk-genes offers moderate diagnostic yield, albeit requiring confirmatory Sanger-sequencing of mutational variants.

**Electronic supplementary material:**

The online version of this article (10.1186/s12872-019-1154-8) contains supplementary material, which is available to authorized users.

## Background

Premature unexpected and unexplained sudden cardiac deaths (SCD) with normal autopsy and toxicology are referred to as sudden arrhythmic death syndrome (SADS) [1, 2], which affects between 0.24 and 0.81 per 100,000 young adults per year in Europe [3]. Contemporaneous epidemiological studies of young SCD (under age 35 years) identify SADS as the commonest certifiable cause in western populations, constituting up to 40% in some series [3–5], although the estimated incidence of SADS is dependent on study design and autopsy protocol [6]. Inherited cardiac ion channel disease, such as the long QT syndrome (LQTS) and Brugada syndrome (BrS) can be identified in up to half of the families of SADS cases [1, 7]. This diagnostic yield of cardiological evaluation, while clinically important in identifying surviving family at risk is limited by incomplete penetrance [7]. Genetic mutation analysis on post-mortem DNA, known as the molecular autopsy, has the potential to identify disease-associated (pathogenic) mutations responsible for SADS, regardless of expressivity; this approach is recommended if provided with appropriate genetic counseling for blood relatives [6, 8].

A yield of 19–26% mutations in LQTS, BrS and catecholaminergic polymorphic ventricular tachycardia (CPVT) risk-genes has been established by 2 large molecular autopsy SADS series from USA [9, 10]; population-based Australasian studies identified yields of up to 27% using exome-based methodology which included analysis of less common arrhythmia and cardiomyopathy-risk genes [4, 11]. Use of formalin-fixed, paraffin-embedded tissue for DNA extraction and limited candidate gene panels have reduced yields in other series [3].

Despite a class IIa recommendation [2, 12], molecular autopsy is infrequently utilized, with monetary cost given as one of the main reasons for failing to do so [13]. Next generation sequencing (NGS) technologies may offer inexpensive methods to overcome financial limitations. Multiplexed PCR-based candidate gene enrichment seen in the Access Array (Fluidigm Corporation, San Francisco) may be appropriate for the moderate genetic target size recommended for molecular autopsy following SADS [14]. This can be coupled with NGS by the HiSeq 2000 (Illumina Inc., San Diego) to provide a low-cost high-throughput candidate gene NGS platform.

We aimed to quantify the diagnostic yield of molecular autopsy identified by this NGS (Fluidigm/Illumina) platform in major candidate risk-genes implicated in LQTS, BrS and CPVT in a cohort of international and multi-ethnic SADS cases. The NGS platform was validated on a population-based Caucasian subset of British SADS cases. We evaluated our NGS platform’s future clinical utility for investigation of SADS.

## Methods

### Study setting

We included cases from two cohorts (British Optimization Cohort and International Cohort) which fulfilled the definition of SADS: SCD aged 1–64 years; no ante-mortem cardiac history; last seen alive and well within 24 h of being found dead; no identified cause of death on toxicological analysis and comprehensive coronial and cardiac autopsy [1, 7, 15]. Demographic characteristics, symptoms, medical and family history of SADS cases were ascertained by direct contact with next of kin where consent was given, and from coroners’ and pathologists’ reports.

### Study cohorts

#### British SADS optimization cohort

Consecutive unexpected Caucasian SCD cases requiring coronial autopsy (*n* = 115) were referred by coroners over 12 months, as part of a previously reported national study (1998 to 1999) [15]. Expert panel confirmed SADS in 56 cases; 46 (82.1%) cases had suitable DNA for inclusion extracted from frozen blood. Whole-genome amplification of DNA with commercial kits was utilized prior to sequencing. Conventional mutation detection with Sanger sequencing and NGS with Fluidigm/Illumina were performed in parallel to optimize the NGS platform on this cohort.

#### International SADS cohort

Unrelated SADS cases (*n* = 174) where frozen blood and/or tissue was available were included; no cases with DNA extracted from neonatal blood spot were included. Suitable DNA was extracted from 151 (86.8%) included cases. This multi-ethnic international cohort was recruited from: population-based coronial series (Cardiac Inherited Disease Registry, Auckland, New Zealand 2000–2009 [11, 16], *n* = 63; SCD Registry, Denmark 2000–2006 [5], *n* = 26); consecutive referrals for autopsy (Royal Brompton Hospital, London 2007–2011, *n* = 19; Sheffield Children’s Hospital 1985–2001, n = 19); and consecutive referrals for familial cardiac evaluation (St George’s or Lewisham Hospitals, London 2009–2011, *n* = 28; Academic Medical Centre, Amsterdam 1995–2011 [17], n = 19). Eleven previously published cases from the New Zealand cohort were diagnosed with LQTS on the basis of variants in LQTS risk-genes following molecular autopsy with Sanger sequencing [11, 16]. Nine of these cases were not included in the analysis (including *RYR2* mutation analysis), while two were included as positive controls. This cohort underwent NGS with the Fluidigm/Illumina platform as described below; indels and structural variants were not evaluated.

### Genetic mutation analysis

The arrhythmia panel consisted of coding exons and intron/exon boundaries of risk-genes for development of LQTS, BrS and CPVT [18–20]: *KCNQ1*, *KCNH2*, *SCN5A*, *KCNE1*, *KCNE2* and *RYR2*.

#### Conventional mutation detection with sanger sequencing

Sequence-specific oligonucleotide primers amplified all LQTS and BrS risk-genes (*KCNQ1*, *KCNH2*, *SCN5A*, *KCNE1*, *KCNE2*) targets before direct sequencing (ABI3730 sequencer, Life Technologies, California). For the CPVT risk-gene *RYR2*, we individually screened 37 prioritized exons (7–9, 13–16, 43–50, 82–84, 87–105) with single strand conformational analysis (SSCA); amplicons demonstrating abnormal conformation were subsequently sequenced selectively with an ABI3730 sequencer (Life Technologies).

#### Next generation sequencing with Fluidigm / Illumina

Custom-designed primer pairs to target all candidate gene exons and splice-sites (including *RYR2*) were designed and optimized for the Fluidigm Access Array [21]. Manufacturer’s protocols (Fluidigm 48.480) were followed to amplify genomic DNA in up to 10-plex PCR reaction wells; subsequently, barcode indexes and sequencing adaptors were added by further PCR. Pooled amplicons were harvested and diluted to prepare unidirectional libraries for 150 base-pair (bp) paired-end sequencing on Illumina HiSeq 2000. Illumina NGS reads were trimmed for base Phred quality control (mean quality in a 30 bp sliding window > 20 and 3′ base quality ≥6) and aligned with BWA (v0.6.1-r112-master) on hg19 human genome reference sequence. Variant-calling was performed using GATK v1.5 (Genome Analysis Toolkit, Broad Institute, Cambridge, USA) [22] without downsampling or removal of PCR duplicates; variants with quality/depth < 5 or depth < 30 were filtered. Identified rare missense exonic or splice-site substitutions were confirmed by conventional PCR amplification and ABI3730 sequencing.

### Analysis of rare genetic variation

Variants were considered rare if non-synonymous or affecting splice sites (±5 bp intron/exon boundary) and demonstrated allele frequency < 0.5% in the ExAC [23] and GO-ESP multiethnic exome databases [24], as identified from the ExAC Browser [25] and The Exome Variant Server [24] respectively. Variants were named according to HGVS recommendations; correct naming was confirmed by use of Mutalyzer [26, 27]. All rare variants were submitted to the in silico predictions servers: PolyPhen [28], SIFT, [29, 30] MutationTaster [31, 32] and MutationAssessor [33] for interpretation. Additionally, classifications were extracted from ClinVar [34]. Conservation was assessed by BLAST and CLUSTAL OMEGA. Finally, rare variants were classified as “pathogenic”, “likely pathogenic”, “benign”, “likely benign” or “uncertain significance” in line with current American College of Medical Genetics (ACMG) guidelines [35]; rare variants were considered disease-associated mutations if classified as pathogenic or likely pathogenic.

### Optimization of NGS

NGS (Fluidigm / Illumina) was compared to the “gold-standard” of conventional PCR and Sanger sequencing, based on LQTS and BrS risk-gene (*KCNQ1*, *KCNH2*, *SCN5A*, *KCNE1*, *KCNE2*) common and rare variation identified in the British SADS Optimization Cohort. This represents the entirety of the population-based UK series of SADS cases. *RYR2* data was not included due to SSCA use and incomplete exon coverage.

### Statistics

Data are expressed as means and standard deviation. Normal approximation interval provides 95% confidence interval CI for yield. Comparison of proportions and means are made with Fisher’s exact and t-test respectively, with *p* < 0.05 considered significant.

## Results

**Fig. 1:**
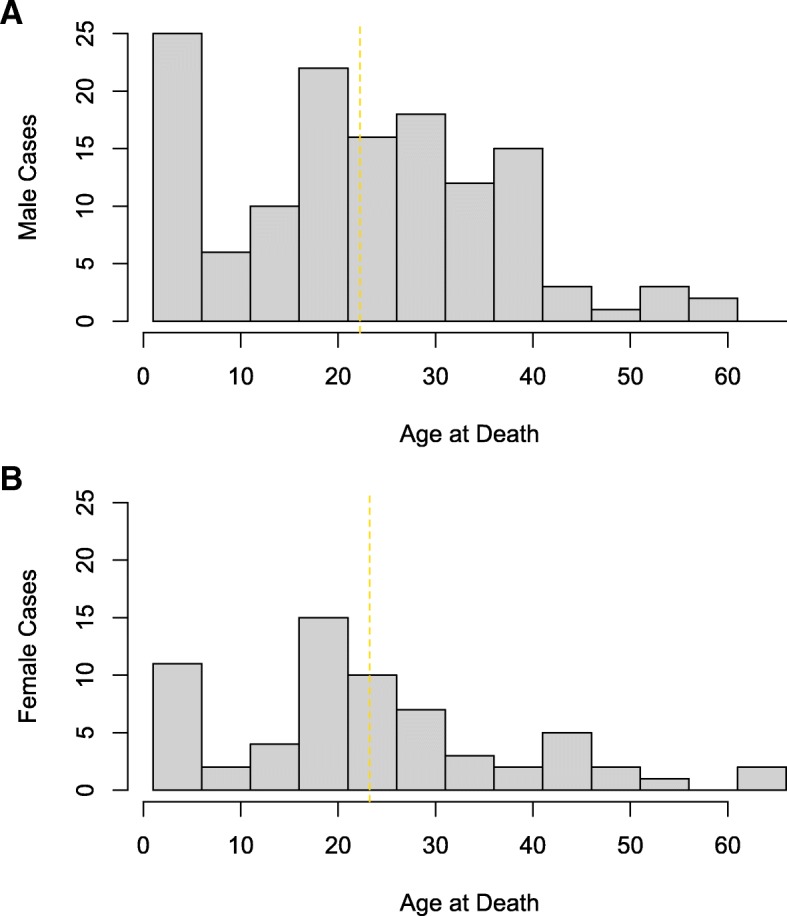
Age Distribution of SADS Cases. Histograms demonstrating bimodal age (years) distribution of all SADS cases included, plotted by gender ([**a**] Male and [**b**] Female). Mean ages indicated by vertical dotted gold lines

**Fig. 2:**
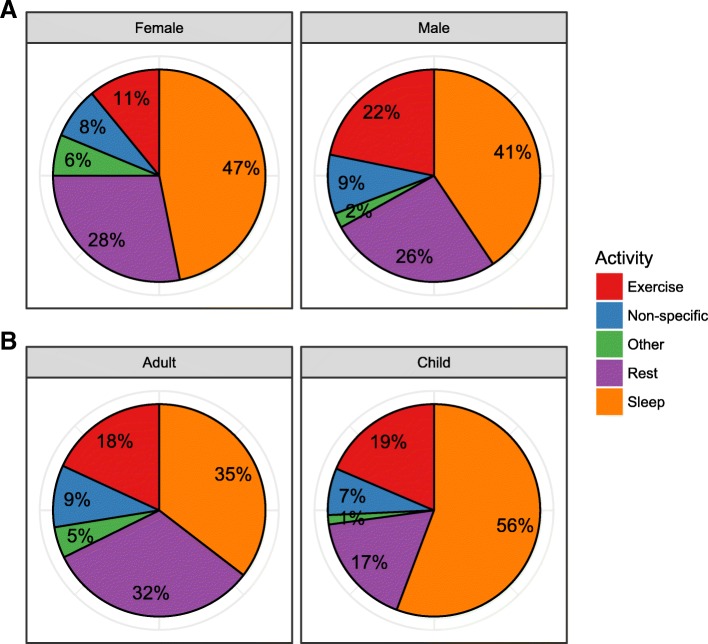
Circumstances of Death amongst SADS Cases. Pie charts demonstrating activity and circumstances at time of death plotted by [**a**] gender and [**b**] age group (children are aged under 18 years)

**Fig. 3:**
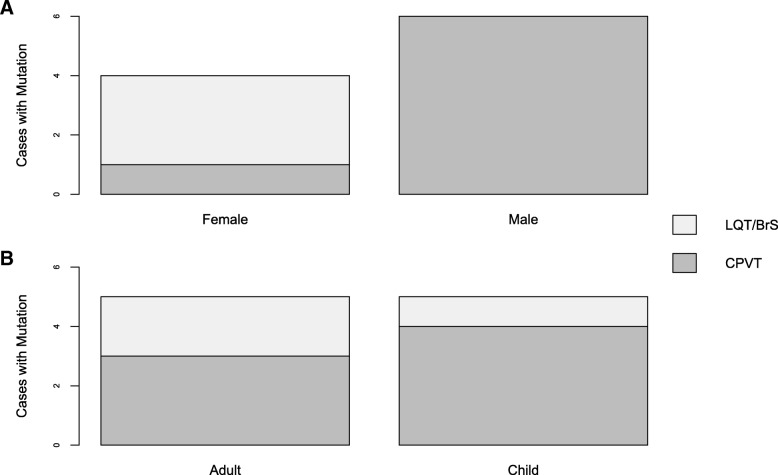
Mutation Carriers amongst SADS cases. Bar charts demonstrating pathogenic and likely pathogenic mutation carriers plotted by [**a**] gender and [**b**] age group (children are aged under 18 years). Abbreviations: BrS = Brugada syndrome; CPVT = catecholaminergic polymorphic ventricular tachycardia; LQTS = long QT syndrome

A total of 197 SADS cases (mean age 22.6 ± 14.4 years, 68% male) were analyzed by combining the British and International cohorts (Fig. 1). There was no difference in proportion of exertional deaths between gender groups or age cohort (*p* = 0.07 and *p* = 1.00 respectively; Fig. 2). Twenty-six different rare variants were identified in 29 cases (Table 1). Two individuals were double heterozygotes, while one rare variant was identified in six unrelated individuals (see Ethnic Variant Analysis below). Detailed variant assessment for ACMG classification of pathogenicity is provided as Additional file 1. A summary of demographic characteristics of carriers of disease-associated mutations is given in Fig. 3.

**Table 1:** Rare Variants identified in SADS Cases

SADS Case ID	Variant	HGMD ID	dbSNP ID	Transcript change	Amino Acid change	ACMG Classification
KCNE1 (NM_000219.5/NP_000210.2)						
314	A		rs75610894	c.142C > T	p.L48F	VUS
KCNE2 (NM_172201.1/NP_751951.1)						
756	B	CM003449	rs2234916	c.22A > G	p.T8A	VUS
KCNH2 (NM_000238.3/NP_000229.1)						
998	C	CM057124	rs199473420	c.211G > C	p.G71R	Likely Pathogenic
217	D	CM002298	rs138776684	c.1039C > T	p.P347S	VUS
411	E			c.2564G > A	p.S855 N	VUS
714	F	CM057119	rs199473017	c.2903C > T	p.P968L	VUS
KCNQ1 (NM_000218.2/NP_000209.2)						
907	G	CM139859	rs794728567	c.969G > A	p.W323*	Likely Pathogenic
1006	H	CM078293	rs12720457	c.1179G > C	p.K393 N	VUS
910	I		rs199472783	c.1379G > A	p.G460D	VUS
1012	J		rs794728542	c.1829C > A	p.T610 N	VUS
RYR2 (NM_001035.2 /NP_001026.2)						
725	K		rs766802574	c.458C > T	p.T153I	Likely Pathogenic
9	L	CM097927	rs794728721	c.1259G > A	p.R420Q	Pathogenic
36	M			c.5248G > A	p.G1750R	Likely Pathogenic
916	N		rs397516546	c.5825 T > G	p.F1942C	VUS
306	O	CM056049	rs794728756	c.7202G > A	p.R2401H	Likely Pathogenic
914	P		rs377763795	c.7458 T > G	p.H2486Q	VUS
708#	Q	CM1515197	rs201500134	c.8162 T > C	p.I2721T	VUS
$ &	R		rs117180147	c.10231-4 T > C		VUS
403	S			c.10681C > G	p.L3561 V	Likely Pathogenic
759	T	CM024349	rs794728777	c.11836G > A	p.G3946S	Pathogenic
38	U	CM148846		c.13823G > A	p.R4608Q	Pathogenic
SCN5A (NM_198056.2/NP_932173.1)						
1034	V	CM033019	rs45620037	c.659C > T	p.T220I	Likely Pathogenic
746&	W	CM034060	rs36210423	c.1715C > A	p.A572D	Likely Benign
727	X		rs72549411	c.2437-5C > A		VUS
738	Y	CM086913	rs41311117	c.6010 T > C	p.F2004 L	VUS
708#	Z	CM004144	rs45489199	c.6016C > G	p.P2006A	VUS

Details of multi-ethnic SADS cohort cases carrying rare variants in major ion channel disease risk-genes: HGMD and dbSNP identifier; transcript and amino acid changes; pathogenicity of variant (ACMG criteria). Abbreviations: VUS = variant of uncertain significance. Annotations: $ = SADS cases 703, 715,722,731,745, 746; #& = double heterozygotes for rare variation

### British SADS optimization cohort

Mean age of the 46 SADS cases was 32.9 ± 14.4 years, comprising 59% males and 28 (61%) non-exertional deaths. Five (11%) families received a clinical diagnosis of genetic disease, as previously reported [1, 15]. Four rare exonic missense variants were identified in four cases; two were disease-associated mutations (variants C and V in Table 1). None of the five cases whose families were diagnosed with inherited heart disease were found to carry any rare variants in the genes sequenced [1, 15]. Clinical diagnostic yield of a disease-related mutation was identified in 4.3% (*n* = 2/46; 95% CI 0.0–10.2%).

### Performance of NGS platform

All samples in the Optimization Cohort had greater than 95% coverage at depth of 10x of the 27049 bp target (*KCNQ1*, *KCNH2*, *SCN5A*, *KCNE1*, *KCNE2*, total 171 amplicons). Mean depth was 3382 reads. Sensitivity for any base change (synonymous or non-synonymous substitution or splice-site variant) for the regions that could be assessed was 87.20% (39 unique variants identified by Sanger sequencing within the target); five amplicons failed amplification. Specificity was 99.99%. Importantly, NGS demonstrated 100% sensitivity for the rare missense variants (*n* = 4). The International Cohort identified the PPV for correctly identifying any rare variation by NGS was 16.0% (27 true positive confirmed by Sanger sequencing of 169 positive calls by NGS platform).

### Ethnic variants

Six New Zealand cases carried the splice-site variant of uncertain significance c.10231-4 T > C (rs117180147) in *RYR2* (NM_001035.2), with five being of Maori ancestry. This variant has no effect on essential splice donor or acceptor bases and is present rarely in East Asians (1.28%), but not seen in Caucasians [24, 36]. No Maori control data was available for comparison.

### Disease-associated mutations

Ten rare variants were pathogenic or likely pathogenic and therefore considered disease-associated (Table 1). The majority of these were missense mutations (*n* = 9), with 1 truncation. This represents 10 SADS cases (5.1%); no disease-associated mutations were seen in more than 1 SADS case. A greater prevalence of mutations amongst female cases was seen with respect to LQTS/BrS risk-mutations (*p* = 0.03), though no significant difference was seen when considering all risk-genes (including *RYR2*, *p* = 0.73). No difference in mutation carrier status was seen between adults and children (*p* = 0.33).

### Pathogenicity of LQTS/BrS risk-mutations

Fifteen rare variants were identified in LQTS/BrS risk-genes, with three disease-associated. Two were reported as previously associated with disease: *KCNH2* (NM_000238.3) N-terminus variant p.G71R associated with LQTS type 2 [37]; *SCN5A* (NM_198056.2) variant p.T220I located in the first transmembrane domain (a region associated with high probability of pathogenicity) [18] which demonstrates in-vitro sodium channel dysfunction [38] and co-segregation with dilated cardiomyopathy and heart block [39]. A novel truncation p.W323* was detected in *KCNQ1* and predicted to cause nonsense-mediated decay [40]. Neither rare variant in *KCNE1* and *KCNE2* were likely to be pathogenic by ACMG criteria.

### Pathogenicity of RYR2 risk-mutations

The 10 rare variants in *RYR2* (excluding the likely ethnic Maori splicing variant c.10231-4 T > C) lie within 10 different exons (7, 14, 37, 38, 47, 49, 54, 74, 88 and 95). Seven were categorised as disease-associated mutations. Five variants (p.R420Q, p.R2401H, p.G3946S and p.R4608Q) were categorised as disease-causing by HGMD, demonstrated 100% mammalian conservation and were previously reported [20, 41]; two other variants (p.T153I, p.G1750R) fulfilled ACMG criteria for likely pathogenicity based on in-silico predictions and conservation data.

### Clinical diagnostic yield

Pathogenic or likely-pathogenic disease-associated mutations in major arrhythmia syndrome risk-genes were identified in 10 of 197 multi-ethnic SADS cases. This represents a diagnostic yield of 5.1% (95% CI 2.0–8.1%).

## Discussion

This study reports on utility of a low-cost high-throughput PCR-based next generation sequencing molecular autopsy in a multi-ethnic internationally recruited series of SADS cases.

### Diagnostic yield of molecular autopsy in SADS

Our multi-ethnic international cohort data support a clinical diagnostic yield for molecular autopsy of SADS cases of up to 5.1% amongst recognized major ion-channelopathy risk-genes, less than that from similarly-sized US studies [9, 10]. The Australasian population-based series of 113 cases demonstrated a comparable yield of 8.8% pathogenic and probably pathogenic mutations utilising NGS [4]. Our prior report (which overlaps with this study cohort) revealed a clinically relevant yield of 10.6% from the common risk-genes studied here, but differs by its use of hybridization-based NGS [17]. Although Wang et al. [10] report on a multi-ethnic SADS population from New York, our study is multi-centre and international. Hence, it remains unclear whether these differences in yield relate to: our complete use of PCR-based NGS as a primary diagnostic genetic methodology; definitions of pathogenicity; ethnicity differences; or local referral bias.

### Potential role of NGS in SADS

NGS has been used for investigation of large SADS cohorts for partial coverage of SCN5A as part of a larger Sanger sequencing study [10], and exome or clinical NGS sequencing in Australasian [4], French [42] and UK [43] series in addition to our previous report [17]. NGS permits use of less DNA and confers greater genetic coverage than conventional technology, which is of particular importance in post-mortem cases where good quality genomic DNA is finite. Moreover, NGS amplification technologies are less labor intensive than Sanger sequencing.

Microfluidic muliplex PCR-based enrichment of candidate genes for NGS (i.e. Fluidigm) offers a balance of throughput and cost-efficiency [21]. Specifically, it offers targeted sequencing which is an order of magnitude cheaper than hybridization capture (e.g. SureSelect, Agilent, Santa Clara, California): approximately $25USD versus $200USD for capture and sequencing.

### RYR2 mutation analysis

Our *RYR2* yield (3.6%, *n* = 7/197) represents over half the clinically-relevant molecular yield, and falls within the range of yields from previously reported large series from USA and Australasia: 3.0% (*n* = 4/133) [10]; 3.5% (n = 4/113) [4]; and 11.6% (*n* = 20/173) [9]. The complete sequencing of *RYR2* with NGS appears to contribute additional yield over limited exon-targeted approaches [9]; 20% (n = 2/10) of our *RYR2* rare variants lay outside the 64 sequenced exons described by Tester et al. [9]; 40% (n = 4/10) would have been missed by a 3-tiered exon mutation analysis strategy for CPVT diagnosis [20].

### Cardiological evaluation of blood relatives

In our population-based British SADS Optimization Cohort, where limited cardiac investigation of families was performed, mutations were not identified in the five SADS cases whose relatives were diagnosed with clinical disease [1]. Molecular autopsy increased our yield of genetic diagnoses (from 10.9 to 15.2%, *n* = 5 to 7). This reinforces the complementary approaches of familial cardiac investigation and molecular autopsy following a SADS death [4, 11, 17].

### Genetic ancestry and the interpretation of genetic results

The *RYR2* splice site variant (c.10231-4 T > C) was present in 5 of the 27 Maori/Polynesian cases. ExAC identifies 1.28% minor allele frequency in East Asians for this variant, and lower frequencies in African and Latino populations. This is plausible as a risk factor for arrhythmic death, similar to that seen with *SCN5A*-S1103Y in the African-American population [44]. Similarly, *SCN5A*-R1193Q has in vitro sodium channel dysfunction and associates with channelopathic disease in Europeans [45], yet the variant is common in Maori, and occurs in over 10% of the Han Chinese [46]. With most studies focusing on ethnic Europeans, there is a risk that other groups may be denied the benefits of genetic diagnosis.

### Clinical implications of the Fluidigm/Illumina NGS platform

Our PPV of 16% mandates approximately 6 variants to be verified by Sanger sequencing for the detection of one true mutation (i.e. five false positives for every true rare variant), with no additional bioinformatics optimization identified to improve this. We believe missed common variants in amplified regions (>10x coverage) of the Optimization Cohort related to preferential amplification of one allele. These findings compare unfavourably with application of this technology in living individuals [14], and could be explained by increased PCR errors encountered in use of lower quality post-mortem DNA. This was supported by appearance of clustering of false positive results in specific cases, suggestive of a DNA sampling issue; no other specific features of false positives were identified to assist in filtering them out. Notably, preferential amplification of a single allele is a recognized limitation of microfluidic PCR-based amplification due to the number of PCR cycles required.

PCR errors are exacerbated by microfluidic technology due to the greater requirement for amplification. Additionally, PCR duplicates were not removed by bioinformatic processing prior to variant calling; removal of duplicates following PCR-based target selection would serve to reduce depth of coverage uniformly to less than 10x, rendering variant calling even less reliable. Given the low likelihood for identical PCR errors with repetition, false positives may be reduced by performing each amplification (and subsequent NGS) in duplicate and only considering variants to be present when identified in both duplicates. However, this method would serve to double the cost per sample of the Fluidigm/Illumina platform in this context. Additionally, the impact on allele amplification and subsequent sensitivity for rare variant identification of this approach is unknown.

Though the financial advantage is being eroded by declining cost of more precise capture technologies such as hybridization [21], this NGS platform may remain useful as an initial screening strategy until economic costs become comparable. In comparison with limited analysis of non-synonymous rare variants in the same risk-genes, no additional clinically relevant variants were identified by hybridization target selection methodology in the 87 cases which overlapped with our prior report [17].

### Study limitations

This study did not analyze small insertions and deletions; such structural rearrangements represent a minority of mutations, making up only 1 in 10 of those with definite LQTS [47, 48]. Copy number variants were not assessed; none were identified in the major risk-genes by Bagnall et al. in their molecular autopsy study [4]. Similarly, we did not address intronic mutations, which are rare in clinical practice, and require large families for co-segregation and in vitro study for confirmation of clinical relevance [49, 50]. GATK v1.5 was used as the updated open-source version available at the time of bioinformatic optimization; we acknowledge that this software has since been improved with respect to variant calling which may further improve the diagnostic precision of the technology.

Clinical data pertaining to surviving blood relatives was not studied systematically; co-segregation did not contribute to novel mutation disease-causing status, which is a significant limitation in the context of forensic post-mortem mutation analysis. Although cardiomyopathy and other arrhythmia syndrome genes were not evaluated, they are likely to contribute less significantly to SADS and pose even greater uncertainty for pathogenicity [4, 17, 43]. The cohort of New Zealand cases was biased by exclusion of a majority (9 of 11) identified LQTS-risk gene variant carriers; these cases did not undergo *RYR2* mutation analysis. Nonetheless, only three would be classified pathogenic or likely pathogenic by modern ACMG criteria, resulting in a minimally affected molecular autopsy yield of 6.3% (*n* = 13/206) if the study cohort was unselected.

## Conclusion

The combination of Fluidigm Access Array with Illumina HiSeq NGS to sequence a selected panel of LQTS/BrS and CPVT risk-genes offers a moderate diagnostic yield of 5.1% amongst SADS cases. Although the low PPV mandates confirmation of mutational variants by Sanger-sequencing, molecular autopsy may also still add significant yield to clinical evaluation of blood relatives.

## Additional file

Additional file 1: Tables and References supporting ACMG classification of variants identified. (PDF 811 kb)

## Data Availability

Rare variant data analysed during this study are included in this published article and its supplementary information files. The raw datasets are not publicly available, but are available from the corresponding author on reasonable request.

## Abbreviations

bp: Base pairs
BrS: Brugada syndrome
CI: Confidence interval
CPVT: Catecholaminergic polymorphic ventricular tachycardia
DNA: Deoxyribonucleic acid
LQTS: Long QT syndrome
NGS: Next generation sequencing
PCR: Polymerase chain reaction
SADS: Sudden arrhythmic death syndrome
SCD: Sudden cardiac death
SSCA: Single strand conformational analysis
