# Supplementary material for: Next-generation sequencing using microfluidic PCR enrichment for molecular autopsy
Source: BMC Cardiovasc Disord. 2019 Jul 23;19:174. doi: 10.1186/s12872-019-1154-8 (PMC6651896; doi:10.1186/s12872-019-1154-8)
Supplement: Supplementary file 1 — Tables and References supporting ACMG classification of variants identified. (PDF 811 kb) [file 12872_2019_1154_MOESM1_ESM.pdf]

Supplemental Material

Cases were screened for genetic variants in six known arrhythmia genes (*KCNE1*, *KCNE2*, *KCNH2*, *KCNQ1*, *RYR2* and *SCN5A*). Twenty-six variants were identified; two individuals were double heterozygotes (Table 1). Variants were named according to HGVS recommendations and Mutalyzer [1, 2] was used to ensure variants were named correctly. The ExAC Brower [3, 4] and The Exome Variant Server [5] were used to retrieve minor allele frequency data from the ExAC and GO-ESP databases, respectively (Table 2). Furthermore, all variants were submitted to the *in silico* predictions servers PolyPhen [6], SIFT [7, 8], MutationTaster [9, 10] and MutationAssessor [11]. Additionally, ClinVar classifications were extracted from ClinVar [12] (Table 2). Conservation was assessed by BLAST and CLUSTAL OMEGA (Table 3). Finally, variants were classified as either “pathogenic”, “likely pathogenic”, “benign”, “likely benign” or “uncertain significance” in line with ACMG guidelines [13] (Table 4).

Table 1: Mutations identified in cohort

Fluidigm	HR-ID	Gene	HGMD ID	dbSNP ID	Transcript	Coding change	Protein	Amino Acid change	References
20LW00392	9	RYR2	CM097927	rs794728721	NM_001035.2	c.1259G>A	NP_001026.2	p.R420Q	[14-19]
20OK02199	36	RYR2			NM_001035.2	c.5248G>A	NP_001026.2	p.G1750R	
20SF02201	38	RYR2	CM148846		NM_001035.2	c.13823G>A	NP_001026.2	p.R4608Q	[19, 20]
20DH02217	217	KCNH2	CM002298	rs138776684	NM_000238.3	c.1039C>T	NP_000229.1	p.P347S	[21-31]
20AM00366	306	RYR2	CM056049	rs794728756	NM_001035.2	c.7202G>A	NP_001026.2	p.R2401H	[15, 32-34]
20AM02224	314	KCNE1		rs75610894	NM_000219.5	c.142C>T	NP_000210.2	p.L48F	
20DE02235	403	RYR2			NM_001035.2	c.10681C>G	NP_001026.2	p.L3561V	[19]
20DE02243	411	KCNH2			NM_000238.3	c.2564G>A	NP_000229.1	p.S855N	
20ML02281	703	RYR2		rs117180147	NM_001035.2	c.10231-4T>C			
20NF02286	708	RYR2	CM1515197	rs201500134	NM_001035.2	c.8162T>C	NP_001026.2	p.I2721T	[35]
		SCN5A	CM004144	rs45489199	NM_198056.2	c.6016C>G	NP_932173.1	p.P2006A	[27, 28, 36-49]
20NX02292	714	KCNH2	CM057119	rs199473017	NM_000238.3	c.2903C>T	NP_000229.1	p.P968L	[21, 25, 50]
20OE02293	715	RYR2		rs117180147	NM_001035.2	c.10231-4T>C			
20PU02300	722	RYR2		rs117180147	NM_001035.2	c.10231-4T>C			
20PJ02303	725	RYR2		rs766802574	NM_001035.2	c.458C>T	NP_001026.2	p.T153I	[19]
20PT02305	727	SCN5A		rs72549411	NM_198056.2	c.2437-5C>A			
20PZ02309	731	RYR2		rs117180147	NM_001035.2	c.10231-4T>C			
20QW02316	738	SCN5A	CM086913	rs41311117	NM_198056.2	c.6010T>C	NP_932173.1	p.F2004L	[36-38, 45, 48, 51-55]
20SF02323	745	RYR2		rs117180147	NM_001035.2	c.10231-4T>C			
20TG02324	746	SCN5A	CM034060	rs36210423	NM_198056.2	c.1715C>A	NP_932173.1	p.A572D	[1, 28, 41, 55-61]
		RYR2		rs117180147	NM_001035.2	c.10231-4T>C			
20LT02271	756	KCNE2	CM003449	rs2234916	NM_172201.1	c.22A>G	NP_751951.1	p.T8A	[1, 3, 5-7, 30, 31, 47, 61-63]
20DN02274	759	RYR2	CM024349	rs794728777	NM_001035.2	c.11836G>A	NP_001026.2	p.G3946S	[12, 15, 19, 64, 65]
20SH00345	907	KCNQ1	CM139859	rs794728567	NM_000218.2	c.969G>A	NP_000209.2	p.W323*	[19]
20SH00347	910	KCNQ1		rs199472783	NM_000218.2	c.1379G>A	NP_000209.2	p.G460D	
20SH00351	914	RYR2		rs377763795	NM_001035.2	c.7458T>G	NP_001026.2	p.H2486Q	
20SH00353	916	RYR2		rs397516546	NM_001035.2	c.5825T>G	NP_001026.2	p.F1942C	
20AS02332	998	KCNH2	CM057124	rs199473420	NM_000238.3	c.211G>C	NP_000229.1	p.G71R	[50, 66]
20SC02339	1006	KCNQ1	CM078293	rs12720457	NM_000218.2	c.1179G>C	NP_000209.2	p.K393N	[1, 21, 24, 25, 50, 67-71]
20BA02345	1012	KCNQ1		rs794728542	NM_000218.2	c.1829C>A	NP_000209.2	p.T610N	
20KP02366	1034	SCN5A	CM033019	rs45620037	NM_198056.2	c.659C>T	NP_932173.1	p.T220I	[9, 21, 23, 34, 46, 47, 53, 54, 72-78]

Table 2: population frequency, in silico predictions and ClinVar classification of genetic variants

Transcript change	Amino Acid change	Minor Allele Frequency (%)				in silico prediction				ClinVar
		ExAC	GO-ESP (ALL)	GO-ESP (AA)	GO-ESP (EA)	Polyphen	MutationTaster	MutationAssessor	Sift	
**KCNE1**										
c.142C>T	p.L48F	0.004	0.015	0.000	0.023	probably damaging	disease causing	medium impact	Damaging	
**KCNE2**										
c.22A>G	p.T8A	0.380	0.492	0.114	0.686	probably damaging	disease causing	medium impact	Damaging	benign
**KCNH2**										
c.211G>C	p.G71R	NA	NA	NA	NA	probably damaging	disease causing	medium impact	Damaging	pathogenic, Congenital long QT syndrome, autosomal dominant
c.1039C>T	p.P347S	0.141	0.046	0.000	0.070	possibly damaging	disease causing	low impact	Tolerated	
c.2564G>A	p.S855N	NA	NA	NA	NA	benign	disease causing	neutral	Tolerated	
c.2903C>T	p.P968L	0.004	0.008	0.000	0.012	benign	polymorphism	neutral	Tolerated	
**KCNQ1**										
c.969G>A	p.W323*	NA	NA	NA	NA		disease causing		Tolerated	pathogenic, …
c.1179G>C	p.K393N	0.110	NA	NA	NA	possibly damaging	disease causing	medium impact	Tolerated	
c.1379G>A	p.G460D	0.006	NA	NA	NA	benign	polymorphism	neutral	Tolerated	variant of uncertain significance, sudden infant death syndrome,
c.1829C>A	p.T610N	NA	NA	NA	NA	possibly damaging	disease causing	low impact	Tolerated	variant of unknown significance
**RYR2**										
c.458C>T	p.T153I	0.002	NA	NA	NA	probably damaging	disease causing	low impact	Damaging	variant of unknown significance
c.1259G>A	p.R420Q	NA	NA	NA	NA	probably damaging	disease causing	medium impact	Damaging	
c.5248G>A	p.G1750R	NA	NA	NA	NA	probably damaging	disease causing	medium impact	Damaging	
c.5825T>G	p.F1942C	0.003	NA	NA	NA	benign	disease causing	medium impact	Tolerated	variant of unknown significance
c.7202G>A	p.R2401H	NA	NA	NA	NA	probably damaging	disease causing	medium impact	Damaging	pathogenic/likely pathogenic, CPVT / cardiovascular phenotype,
c.7458T>G	p.H2486Q	0.001	0.008	0.000	0.012	possibly damaging	disease causing	low impact	Tolerated	
c.8162T>C	p.I2721T	0.057	0.052	0.056	0.050	possibly damaging	disease causing	low impact	Tolerated	variant of unknown significance, CPVT
c.10231-4T>C		0.115	0.017	0.055	0.000	unknown				
c.10681C>G	p.L3561V	NA	NA	NA	NA	possibly damaging	disease causing	medium impact	Damaging	
c.11836G>A	p.G3946S	NA	NA	NA	NA	probably damaging	disease causing	medium impact	Damaging	pathogenic, …
c.13823G>A	p.R4608Q	NA	NA	NA	NA	probably damaging	disease causing	medium impact	Tolerated	
**SCN5A**										
c.659C>T	p.T220I	0.101	0.032	0.000	0.048	probably damaging	disease causing	medium impact	Damaging	Benign/Likely benign/Pathogenic/Uncertain significance, SSS/DCM/BrS
c.1715C>A	p.A572D	0.430	0.169	0.025	0.239	benign	polymorphism	low impact	Tolerated	benign/likely benign, LQT/BRS/SSS/LVNC/PFHB/DCM
c.6010T>C	p.F2004L	0.202	NA	NA	NA	benign	polymorphism	neutral	Tolerated	benign/likely benign/variant of uncertain significance, BrS/LQT
c.6016C>G	p.P2006A	0.134	0.113	0.025	0.155	benign	polymorphism	neutral	Tolerated	likely benign/uncertain significance, BrS
c.2437-5C>A		0.035	0.054	0.023	0.070	unknown				

Table 3: HGMD classification and conservation summary

Transcript change	Amino Acid change	HGMD Variant Classification	Conservation
**KCNE1**			
c.142C>T	p.L48F		p.L48F - "L" is 94% conserved among 17 species: 100% conserved among 14 mammalian species and 67% conserved among 3 non-mammalian species. Carp carry a "C" at this residue. The 21 amino acids surrounding the residue were 87 % conserved in 14 mammalian species and 45 % conserved in 3 non-mammalian species.
**KCNE2**			
c.22A>G	p.T8A	Disease-associated polymorphism	p.T8A -" T" is 100% conserved across 60 species. The 21 amino acids surrounding this residue are 79% conserved across 56 mammalian species and 56% conserved across 4 non-mammalian species.
**KCNH2**			
c.211G>C	p.G71R	Disease causing mutation	p.G71R - "G" is 100% conserved across 45 species. The 21 amino acids surrounding this residue are 100% conserved in 40 mammalian species and 86% conserved among 6 non-mammalian species.
c.1039C>T	p.P347S	Disease causing mutation	p.P347S - "P" is conserved 100% across 48 mammalian species, this residue is not conserved in 8 non-mammalian species, and however, no species carried an "S". The 21 amino acids surrounding this residue are 99% conserved in 48 mammalian species and 65 % conserved in 8 non-mammalian species
c.2564G>A	p.S855N		p.S855N - "S" is 65% conserved among 20 species: 100% conserved among 10 mammalian species and 30% conserved among 10 non-mammalian species. Carp, Zebrafish, Chicken, Croaker, Pufferfish, Arowana fish, and King Cobra carry an "N" at this residue. The 21 amino acids surrounding the residue were 100 % conserved in 10 mammalian species and 89 % conserved in 10 non-mammalian species.
c.2903C>T	p.P968L	Disease causing mutation	p.P968L - "P" is 100% conserved in mammalian species and the 21 amino acids surrounding the residue are 87 % conserved in 28 mammalian species
**KCNQ1**			
c.969G>A	p.W323*	Disease causing mutation	
c.1179G>C	p.K393N	Disease causing mutation	p.K393N - "K" is 100% conserved in 17 non-mammalian species and 98% conserved in 48 mammalian species. Domestic Cat carries "N" at this position. The 21 amino acids surrounding this residue are 85% conserved in 48 mammalian species and 80% conserved in 17 non-mammalian species.
c.1379G>A	p.G460D		p.G460D - "G" is 30% conserved among 30 species (81% among 11 mammalian species), no species carried a D at this position. The 21 amino acids surrounding this residue are 49% conserved in 19 non-mammalian species and 84% conserved in 11 mammalian species.
c.1829C>A	p.T610N		p.T610N - "T" 91% conserved in 12 species (100% among 9 mammalian species). The 21 amino acids surrounding this residue were 92% conserved among 9 mammalian species and 52 % conserved among non-mammalian species.
**RYR2**			
c.458C>T	p.T153I		p.T153I - "T" is conserved in 77% of 22 species (90% among 10 mammalian species), no species carried and "I" at this position. The 21 amino acids surrounding the residue were 97.6% in 12 non-mammalian species and 99.5% in 10 mammalian species.
c.1259G>A	p.R420Q	Disease causing mutation	p.R420Q - "R" is 100% conserved in all species identified by BLAST search. The 21 amino acids surrounding this residue were 100% conserved in 57 mammalian species ad 92% conserved in 20 non-mammalian species.
c.5248G>A	p.G1750R		p.G1750R - "G" is conserved 100% in 21 species. The 21 amino acids surrounding this residue were 96.1% conserved in 11 non-mammalian species and 94.3% conserved in 10 mammalian species.
c.5825T>G	p.F1942C		p.F1942C - "F" is conserved 100% in 23 species. The 21 amino acids surrounding this residue were 92.3% conserved in 13 non-mammalian species and 99% conserved in 10 mammalian species.
c.7202G>A	p.R2401H	Disease causing mutation	p.R2401H - "R" is 100% conserved in all species identified by BLAST search. The 21 amino acids surrounding the residue were 97% conserved in 56 mammalian species and 94 % conserved in 24 non-mammalian species including Acorn worm
c.7458T>G	p.H2486Q		p.H2486Q - "H" is conserved 100% in 22 species. The 21 amino acids surrounding this residue were 94.8%% conserved in 12non-mammalian species and 99.5% conserved in 10 mammalian species.
c.8162T>C	p.I2721T	Disease causing mutation?	p.I2721T -"I" is 100% conserved in all species identified by BLAST search. The 21 amino acids surrounding the residue were 99% conserved in 54 mammalian species and 97 % conserved in 21 non-mammalian species
c.10231-4T>C			
c.10681C>G	p.L3561V		p.L3561V - "L" is conserved 100 % in 20 species. The 21 amino acids surrounding this residue were 63.2 % conserved in 10 non-mammalian species and 68.3 % conserved in mammalian species.
c.11836G>A	p.G3946S	Disease causing mutation	p.G3946S - "G" is 100% conserved in all species identified by BLAST search. The 21 amino acids surrounding the residue were 100 % conserved in 57 mammalian species and 100 % conserved in 22 non-mammalian species including Vase tunicate.
c.13823G>A	p.R4608Q	Disease causing mutation	p.R4608Q - "R" is 100% conserved in all species identified by BLAST search. The 21 amino acids surrounding the residue were 100 % conserved in 57 mammalian species and 99 % conserved in 22 non-mammalian species including Vase tunicate.
**SCN5A**			

c.659C>T	p.T220I	Disease causing mutation	p.T220I -"T" is 100% conserved in all species identified by BLAST search. The 21 amino acids surrounding the residue were 96 % conserved in 56 mammalian species and 91 % conserved in 7 non-mammalian species.
c.1715C>A	p.A572D	Disease causing mutation?	p.A572D - "A" is 73% conserved among 52 mammalian species, the surrounding 21 amino acids are 94% conserved. No non-mammalian species were identified in a BLAST search.
c.2437-5C>A			
c.6010T>C	p.F2004L	Disease causing mutation?	p.F2004L - "F" is 90% conserved among 51 mammalian species, the surrounding 21 amino acids are 96% conserved. No non-mammalian species were identified in a BLAST search. Long tailed chinchilla and dog, both carry "L" at this position.
c.6016C>G	p.P2006A	Disease causing mutation?	p.P2006A - "P" is 84% conserved among 51 mammalian species, the surrounding 21 amino acids are 91% conserved. No non-mammalian species were identified in a BLAST search. Domestic guinea pig, domestic cat, Brandt's bat, mouse-eared bat and little brown bat all carry "A" at this position.

Table 4: Variant classification

HGMD ID	dbSNP ID	Transcript change	Amino Acid change	Classification
**KCNE1 (NM_000219.5/NP_000210.2)**				
	rs75610894	c.142C>T	p.L48F	Uncertain significance
**KCNE2 (NM_172201.1/NP_751951.1)**				
CM003449	rs2234916	c.22A>G	p.T8A	Uncertain significance
**KCNH2 (NM_000238.3/NP_000229.1)**				
CM057124	rs199473420	c.211G>C	p.G71R	Likely pathogenic
CM002298	rs138776684	c.1039C>T	p.P347S	Uncertain significance
		c.2564G>A	p.S855N	Uncertain significance
CM057119	rs199473017	c.2903C>T	p.P968L	Uncertain significance
**KCNQ1 (NM_000218.2/NP_000209.2)**				
CM139859	rs794728567	c.969G>A	p.W323*	Likely pathogenic
CM078293	rs12720457	c.1179G>C	p.K393N	Uncertain significance
	rs199472783	c.1379G>A	p.G460D	Uncertain significance
	rs794728542	c.1829C>A	p.T610N	Uncertain significance
**RYR2 (NM_001035.2 /NP_001026.2)**				
	rs766802574	c.458C>T	p.T153I	Likely pathogenic
CM097927	rs794728721	c.1259G>A	p.R420Q	Pathogenic
		c.5248G>A	p.G1750R	Likely pathogenic
	rs397516546	c.5825T>G	p.F1942C	Uncertain significance
CM056049	rs794728756	c.7202G>A	p.R2401H	Likely pathogenic
	rs377763795	c.7458T>G	p.H2486Q	Uncertain significance
CM1515197	rs201500134	c.8162T>C	p.I2721T	Uncertain significance
	rs117180147	c.10231-4T>C		Uncertain significance
		c.10681C>G	p.L3561V	Likely pathogenic
CM024349	rs794728777	c.11836G>A	p.G3946S	Pathogenic
CM148846		c.13823G>A	p.R4608Q	Pathogenic
**SCN5A (NM_198056.2/NP_932173.1)**				
CM033019	rs45620037	c.659C>T	p.T220I	Likely pathogenic
CM034060	rs36210423	c.1715C>A	p.A572D	Likely benign
	rs72549411	c.2437-5C>A		Uncertain significance
CM086913	rs41311117	c.6010T>C	p.F2004L	Uncertain significance
CM004144	rs45489199	c.6016C>G	p.P2006A	Uncertain significance

References

[1] Mank-Seymour AR, Richmond JL, Wood LS, Reynolds JM, Fan YT, Warnes GR, Milos PM, Thompson JF. Association of torsades de pointes with novel and known single nucleotide polymorphisms in long QT syndrome genes. Am Heart J 2006;152:1116-22.
[2] Wildeman M, van Ophuizen E, den Dunnen JT, Taschner PE. Improving sequence variant descriptions in mutation databases and literature using the Mutalyzer sequence variation nomenclature checker. Hum Mutat 2008;29:6-13.
[3] Aydin A, Bahring S, Dahm S, Guenther UP, Uhlmann R, Busjahn A, Luft FC. Single nucleotide polymorphism map of five long-QT genes. J Mol Med (Berl) 2005;83:159-65.
[4] Lek M, Karczewski KJ, Minikel EV, Samocha KE, Banks E, Fennell T, O'Donnell-Luria AH, Ware JS, Hill AJ, Cummings BB, Tukiainen T, Birnbaum DP, Kosmicki JA, Duncan LE, Estrada K, Zhao F, Zou J, Pierce-Hoffman E, Berghout J, Cooper DN, Deflaux N, DePristo M, Do R, Flannick J, Fromer M, Gauthier L, Goldstein J, Gupta N, Howrigan D, Kiezun A, Kurki MI, Moonshine AL, Natarajan P, Orozco L, Peloso GM, Poplin R, Rivas MA, Ruano-Rubio V, Rose SA, Ruderfer DM, Shakir K, Stenson PD, Stevens C, Thomas BP, Tiao G, Tusie-Luna MT, Weisburd B, Won HH, Yu D, Altshuler DM, Ardissino D, Boehnke M, Danesh J, Donnelly S, Elosua R, Florez JC, Gabriel SB, Getz G, Glatt SJ, Hultman CM, Kathiresan S, Laakso M, McCarroll S, McCarthy MI, McGovern D, McPherson R, Neale BM, Palotie A, Purcell SM, Saleheen D, Scharf JM, Sklar P, Sullivan PF, Tuomilehto J, Tsuang MT, Watkins HC, Wilson JG, Daly MJ, MacArthur DG, Exome Aggregation C. Analysis of protein-coding genetic variation in 60,706 humans. Nature 2016;536:285-91.
[5] Sesti F, Abbott GW, Wei J, Murray KT, Saksena S, Schwartz PJ, Priori SG, Roden DM, George AL, Jr., Goldstein SA. A common polymorphism associated with antibiotic-induced cardiac arrhythmia. Proc Natl Acad Sci U S A 2000;97:10613-8.
[6] Hedley PL, Haundrup O, Andersen PS, Aidt FH, Jensen M, Moolman-Smook JC, Bundgaard H, Christiansen M. The KCNE genes in hypertrophic cardiomyopathy: a candidate gene study. J Negat Results Biomed 2011;10:12.
[7] Larsen LA, Andersen PS, Kanters J, Svendsen IH, Jacobsen JR, Vuust J, Wettrell G, Tranebjaerg L, Bathen J, Christiansen M. Screening for mutations and polymorphisms in the genes KCNH2 and KCNE2 encoding the cardiac HERG/MiRP1 ion channel: implications for acquired and congenital long Q-T syndrome. Clin Chem 2001;47:1390-5.
[8] Kumar P, Henikoff S, Ng PC. Predicting the effects of coding non-synonymous variants on protein function using the SIFT algorithm. Nat Protoc 2009;4:1073-81.
[9] Olson TM, Michels VV, Ballew JD, Reyna SP, Karst ML, Herron KJ, Horton SC, Rodeheffer RJ, Anderson JL. Sodium channel mutations and susceptibility to heart failure and atrial fibrillation. JAMA 2005;293:447-54.
[10] Schwarz JM, Cooper DN, Schuelke M, Seelow D. MutationTaster2: mutation prediction for the deep-sequencing age. Nat Methods 2014;11:361-2.
[11] Millat G, Chevalier P, Restier-Miron L, Da Costa A, Bouvagnet P, Kugener B, Fayol L, Gonzalez Armengod C, Oddou B, Chanavat V, Froidefond E, Perraudin R, Rousson R, Rodriguez-Lafrasse C. Spectrum of pathogenic mutations and associated polymorphisms in a cohort of 44 unrelated patients with long QT syndrome. Clin Genet 2006;70:214-27.
[12] Kawamura M, Ohno S, Naiki N, Nagaoka I, Dochi K, Wang Q, Hasegawa K, Kimura H, Miyamoto A, Mizusawa Y, Itoh H, Makiyama T, Sumitomo N, Ushinohama H, Oyama K, Murakoshi N, Aonuma K, Horigome H, Honda T, Yoshinaga M, Ito M, Horie M. Genetic background of catecholaminergic polymorphic ventricular tachycardia in Japan. Circ J 2013;77:1705-13.
[13] Richards S, Aziz N, Bale S, Bick D, Das S, Gastier-Foster J, Grody WW, Hegde M, Lyon E, Spector E, Voelkerding K, Rehm HL, Committee ALQA. Standards and guidelines for the interpretation of sequence variants: a joint consensus recommendation of the American College of Medical Genetics and Genomics and the Association for Molecular Pathology. Genet Med 2015;17:405-24.
[14] Domingo D, Neco P, Fernandez-Pons E, Zissimopoulos S, Molina P, Olague J, Suarez-Mier MP, Lai FA, Gomez AM, Zorio E. Non-ventricular, Clinical, and Functional Features of the RyR2(R420Q) Mutation

Causing Catecholaminergic Polymorphic Ventricular Tachycardia. Rev Esp Cardiol (Engl Ed) 2015;68:398-407.
[15] Jabbari J, Jabbari R, Nielsen MW, Holst AG, Nielsen JB, Haunso S, Tfelt-Hansen J, Svendsen JH, Olesen MS. New exome data question the pathogenicity of genetic variants previously associated with catecholaminergic polymorphic ventricular tachycardia. Circ Cardiovasc Genet 2013;6:481-9.
[16] Kimlicka L, Tung CC, Carlsson AC, Lobo PA, Yuchi Z, Van Petegem F. The cardiac ryanodine receptor N-terminal region contains an anion binding site that is targeted by disease mutations. Structure 2013;21:1440-9.
[17] Medeiros-Domingo A, Bhuiyan ZA, Tester DJ, Hofman N, Bikker H, van Tintelen JP, Mannens MM, Wilde AA, Ackerman MJ. The RYR2-encoded ryanodine receptor/calcium release channel in patients diagnosed previously with either catecholaminergic polymorphic ventricular tachycardia or genotype negative, exercise-induced long QT syndrome: a comprehensive open reading frame mutational analysis. J Am Coll Cardiol 2009;54:2065-74.
[18] Novak A, Barad L, Lorber A, Gherghiceanu M, Reiter I, Eisen B, Eldor L, Itskovitz-Eldor J, Eldar M, Arad M, Binah O. Functional abnormalities in iPSC-derived cardiomyocytes generated from CPVT1 and CPVT2 patients carrying ryanodine or calsequestrin mutations. J Cell Mol Med 2015;19:2006-18.
[19] Lahrouchi N, Raju H, Lodder EM, Papatheodorou E, Ware JS, Papadakis M, Tadros R, Cole D, Skinner JR, Crawford J, Love DR, Pua CJ, Soh BY, Bhalshankar JD, Govind R, Tfelt-Hansen J, Winkel BG, van der Werf C, Wijeyeratne YD, Mellor G, Till J, Cohen MC, Tome-Esteban M, Sharma S, Wilde AAM, Cook SA, Bezzina CR, Sheppard MN, Behr ER. Utility of Post-Mortem Genetic Testing in Cases of Sudden Arrhythmic Death Syndrome. J Am Coll Cardiol 2017;69:2134-45.
[20] Wong LC, Roses-Noguer F, Till JA, Behr ER. Cardiac evaluation of pediatric relatives in sudden arrhythmic death syndrome: a 2-center experience. Circ Arrhythm Electrophysiol 2014;7:800-6.
[21] Amendola LM, Dorschner MO, Robertson PD, Salama JS, Hart R, Shirts BH, Murray ML, Tokita MJ, Gallego CJ, Kim DS, Bennett JT, Crosslin DR, Ranchalis J, Jones KL, Rosenthal EA, Jarvik ER, Itsara A, Turner EH, Herman DS, Schleit J, Burt A, Jamal SM, Abrudan JL, Johnson AD, Conlin LK, Dulik MC, Santani A, Metterville DR, Kelly M, Foreman AK, Lee K, Taylor KD, Guo X, Crooks K, Kiedrowski LA, Raffel LJ, Gordon O, Machini K, Desnick RJ, Biesecker LG, Lubitz SA, Mulchandani S, Cooper GM, Joffe S, Richards CS, Yang Y, Rotter JI, Rich SS, O'Donnell CJ, Berg JS, Spinner NB, Evans JP, Fullerton SM, Leppig KA, Bennett RL, Bird T, Sybert VP, Grady WM, Tabor HK, Kim JH, Bamshad MJ, Wilfond B, Motulsky AG, Scott CR, Pritchard CC, Walsh TD, Burke W, Raskind WH, Byers P, Hisama FM, Rehm H, Nickerson DA, Jarvik GP. Actionable exomic incidental findings in 6503 participants: challenges of variant classification. Genome Res 2015;25:305-15.
[22] Anderson CL, Delisle BP, Anson BD, Kilby JA, Will ML, Tester DJ, Gong Q, Zhou Z, Ackerman MJ, January CT. Most LQT2 mutations reduce Kv11.1 (hERG) current by a class 2 (trafficking-deficient) mechanism. Circulation 2006;113:365-73.
[23] Dorschner MO, Amendola LM, Turner EH, Robertson PD, Shirts BH, Gallego CJ, Bennett RL, Jones KL, Tokita MJ, Bennett JT, Kim JH, Rosenthal EA, Kim DS, National Heart L, Blood Institute Grand Opportunity Exome Sequencing P, Tabor HK, Bamshad MJ, Motulsky AG, Scott CR, Pritchard CC, Walsh T, Burke W, Raskind WH, Byers P, Hisama FM, Nickerson DA, Jarvik GP. Actionable, pathogenic incidental findings in 1,000 participants' exomes. Am J Hum Genet 2013;93:631-40.
[24] Ghouse J, Have CT, Weeke P, Bille Nielsen J, Ahlberg G, Balslev-Harder M, Appel EV, Skaaby T, Olesen SP, Grarup N, Linneberg A, Pedersen O, Haunso S, Hastrup Svendsen J, Hansen T, Kanters JK, Salling Olesen M. Rare genetic variants previously associated with congenital forms of long QT syndrome have little or no effect on the QT interval. Eur Heart J 2015;36:2523-9.
[25] Giudicessi JR, Kapplinger JD, Tester DJ, Alders M, Salisbury BA, Wilde AA, Ackerman MJ. Phylogenetic and physicochemical analyses enhance the classification of rare nonsynonymous single nucleotide variants in type 1 and 2 long-QT syndrome. Circ Cardiovasc Genet 2012;5:519-28.
[26] Jou CJ, Barnett SM, Bian JT, Weng HC, Sheng X, Tristani-Firouzi M. An in vivo cardiac assay to determine the functional consequences of putative long QT syndrome mutations. Circ Res 2013;112:826-30.

[27] Novotny T, Kadlecova J, Raudenska M, Bittnerova A, Andrsova I, Florianova A, Vasku A, Neugebauer P, Kozak M, Sepsi M, Krivan L, Gaillyova R, Spinar J. Mutation analysis ion channel genes ventricular fibrillation survivors with coronary artery disease. Pacing Clin Electrophysiol 2011;34:742-9.
[28] Refsgaard L, Holst AG, Sadjadieh G, Haunso S, Nielsen JB, Olesen MS. High prevalence of genetic variants previously associated with LQT syndrome in new exome data. Eur J Hum Genet 2012;20:905-8.
[29] Splawski I, Shen J, Timothy KW, Lehmann MH, Priori S, Robinson JL, Moss AJ, Schwartz PJ, Towbin JA, Vincent GM, Keating MT. Spectrum of mutations in long-QT syndrome genes. KVLQT1, HERG, SCN5A, KCNE1, and KCNE2. Circulation 2000;102:1178-85.
[30] Paulussen AD, Gilissen RA, Armstrong M, Doevendans PA, Verhasselt P, Smeets HJ, Schulze-Bahr E, Haverkamp W, Breithardt G, Cohen N, Aerssens J. Genetic variations of KCNQ1, KCNH2, SCN5A, KCNE1, and KCNE2 in drug-induced long QT syndrome patients. J Mol Med (Berl) 2004;82:182-8.
[31] Jongbloed R, Marcelis C, Velter C, Doevendans P, Geraedts J, Smeets H. DHPLC analysis of potassium ion channel genes in congenital long QT syndrome. Hum Mutat 2002;20:382-91.
[32] Aizawa Y, Ueda K, Komura S, Washizuka T, Chinushi M, Inagaki N, Matsumoto Y, Hayashi T, Takahashi M, Nakano N, Yasunami M, Kimura A, Hiraoka M, Aizawa Y. A novel mutation in FKBP12.6 binding region of the human cardiac ryanodine receptor gene (R2401H) in a Japanese patient with catecholaminergic polymorphic ventricular tachycardia. Int J Cardiol 2005;99:343-5.
[33] Roux-Buisson N, Egea G, Denjoy I, Guicheney P, Lunardi J. Germline and somatic mosaicism for a mutation of the ryanodine receptor type 2 gene: implication for genetic counselling and patient caring. Europace 2011;13:130-2.
[34] Walsh R, Peters NS, Cook SA, Ware JS. Paralogue annotation identifies novel pathogenic variants in patients with Brugada syndrome and catecholaminergic polymorphic ventricular tachycardia. J Med Genet 2014;51:35-44.
[35] Hertz CL, Christiansen SL, Ferrero-Miliani L, Fordyce SL, Dahl M, Holst AG, Ottesen GL, Frank-Hansen R, Bundgaard H, Morling N. Next-generation sequencing of 34 genes in sudden unexplained death victims in forensics and in patients with channelopathic cardiac diseases. Int J Legal Med 2015;129:793-800.
[36] Ackerman MJ, Splawski I, Makielski JC, Tester DJ, Will ML, Timothy KW, Keating MT, Jones G, Chadha M, Burrow CR, Stephens JC, Xu C, Judson R, Curran ME. Spectrum and prevalence of cardiac sodium channel variants among black, white, Asian, and Hispanic individuals: implications for arrhythmogenic susceptibility and Brugada/long QT syndrome genetic testing. Heart Rhythm 2004;1:600-7.
[37] Andreasen C, Refsgaard L, Nielsen JB, Sajadieh A, Winkel BG, Tfelt-Hansen J, Haunso S, Holst AG, Svendsen JH, Olesen MS. Mutations in genes encoding cardiac ion channels previously associated with sudden infant death syndrome (SIDS) are present with high frequency in new exome data. Can J Cardiol 2013;29:1104-9.
[38] Kapplinger JD, Tester DJ, Alders M, Benito B, Berthet M, Brugada J, Brugada P, Fressart V, Guerchicoff A, Harris-Kerr C, Kamakura S, Kyndt F, Koopmann TT, Miyamoto Y, Pfeiffer R, Pollevick GD, Probst V, Zumhagen S, Vatta M, Towbin JA, Shimizu W, Schulze-Bahr E, Antzelevitch C, Salisbury BA, Guicheney P, Wilde AA, Brugada R, Schott JJ, Ackerman MJ. An international compendium of mutations in the SCN5A-encoded cardiac sodium channel in patients referred for Brugada syndrome genetic testing. Heart Rhythm 2010;7:33-46.
[39] Krahn AD, Healey JS, Chauhan V, Birnie DH, Simpson CS, Champagne J, Gardner M, Sanatani S, Exner DV, Klein GJ, Yee R, Skanes AC, Gula LJ, Gollob MH. Systematic assessment of patients with unexplained cardiac arrest: Cardiac Arrest Survivors With Preserved Ejection Fraction Registry (CASPER). Circulation 2009;120:278-85.
[40] Lieve KV, Williams L, Daly A, Richard G, Bale S, Macaya D, Chung WK. Results of genetic testing in 855 consecutive unrelated patients referred for long QT syndrome in a clinical laboratory. Genet Test Mol Biomarkers 2013;17:553-61.
[41] Maxwell KN, Hart SN, Vijai J, Schrader KA, Slavin TP, Thomas T, Wubbenhorst B, Ravichandran V, Moore RM, Hu C, Guidugli L, Wenz B, Domchek SM, Robson ME, Szabo C, Neuhausen SL, Weitzel JN, Offit K,

Couch FJ, Nathanson KL. Evaluation of ACMG-Guideline-Based Variant Classification of Cancer Susceptibility and Non-Cancer-Associated Genes in Families Affected by Breast Cancer. Am J Hum Genet 2016;98:801-17.
[42] Priori SG, Napolitano C, Schwartz PJ, Bloise R, Crotti L, Ronchetti E. The elusive link between LQT3 and Brugada syndrome: the role of flecainide challenge. Circulation 2000;102:945-7.
[43] Shinlapawittayatorn K, Du XX, Liu H, Ficker E, Kaufman ES, Deschenes I. A common SCN5A polymorphism modulates the biophysical defects of SCN5A mutations. Heart Rhythm 2011;8:455-62.
[44] Skinner JR, Crawford J, Smith W, Aitken A, Heaven D, Evans CA, Hayes I, Neas KR, Stables S, Koelmeyer T, Denmark L, Vuletic J, Maxwell F, White K, Yang T, Roden DM, Leren TP, Shelling A, Love DR, Cardiac Inherited Disease Group New Z. Prospective, population-based long QT molecular autopsy study of postmortem negative sudden death in 1 to 40 year olds. Heart Rhythm 2011;8:412-9.
[45] Wang DW, Desai RR, Crotti L, Arnestad M, Insolia R, Pedrazzini M, Ferrandi C, Vege A, Rognum T, Schwartz PJ, George AL, Jr. Cardiac sodium channel dysfunction in sudden infant death syndrome. Circulation 2007;115:368-76.
[46] Norton N, Robertson PD, Rieder MJ, Zuchner S, Rampersaud E, Martin E, Li D, Nickerson DA, Hershberger RE, National Heart L, Blood Institute GOESP. Evaluating pathogenicity of rare variants from dilated cardiomyopathy in the exome era. Circ Cardiovasc Genet 2012;5:167-74.
[47] Sudandiradoss C, Sethumadhavan R. In silico investigations on functional and haplotype tag SNPs associated with congenital long QT syndromes (LQTSs). Genomic Med 2008;2:55-67.
[48] Arnestad M, Crotti L, Rognum TO, Insolia R, Pedrazzini M, Ferrandi C, Vege A, Wang DW, Rhodes TE, George AL, Jr., Schwartz PJ. Prevalence of long-QT syndrome gene variants in sudden infant death syndrome. Circulation 2007;115:361-7.
[49] Hofman-Bang J, Behr ER, Hedley P, Tfelt-Hansen J, Kanters JK, Haunsoe S, McKenna WJ, Christiansen M. High-efficiency multiplex capillary electrophoresis single strand conformation polymorphism (multi-CE-SSCP) mutation screening of SCN5A: a rapid genetic approach to cardiac arrhythmia. Clin Genet 2006;69:504-11.
[50] Napolitano C, Priori SG, Schwartz PJ, Bloise R, Ronchetti E, Nastoli J, Bottelli G, Cerrone M, Leonardi S. Genetic testing in the long QT syndrome: development and validation of an efficient approach to genotyping in clinical practice. JAMA 2005;294:2975-80.
[51] Bebarova M, O'Hara T, Geelen JL, Jongbloed RJ, Timmermans C, Arens YH, Rodriguez LM, Rudy Y, Volders PG. Subepicardial phase 0 block and discontinuous transmural conduction underlie right precordial ST-segment elevation by a SCN5A loss-of-function mutation. Am J Physiol Heart Circ Physiol 2008;295:H48-58.
[52] Bokeria LA, Revishvili A, Pronicheva IV, Zakliaz'minskaia EV, Poliakov AV. [The clinical variability of and approaches to treatment of life-threatening ventricular arrhythmias caused by SCN5A gene mutations]. Vestn Ross Akad Med Nauk 2007:3-11.
[53] Olesen MS, Yuan L, Liang B, Holst AG, Nielsen N, Nielsen JB, Hedley PL, Christiansen M, Olesen SP, Haunso S, Schmitt N, Jespersen T, Svendsen JH. High prevalence of long QT syndrome-associated SCN5A variants in patients with early-onset lone atrial fibrillation. Circ Cardiovasc Genet 2012;5:450-9.
[54] Risgaard B, Jabbari R, Refsgaard L, Holst AG, Haunso S, Sadjadieh A, Winkel BG, Olesen MS, Tfelt-Hansen J. High prevalence of genetic variants previously associated with Brugada syndrome in new exome data. Clin Genet 2013;84:489-95.
[55] Albert CM, Nam EG, Rimm EB, Jin HW, Hajjar RJ, Hunter DJ, MacRae CA, Ellinor PT. Cardiac sodium channel gene variants and sudden cardiac death in women. Circulation 2008;117:16-23.
[56] Clendenen N, Cannon AD, Porter S, Robards CB, Parker AS, Clendenen SR. Whole-exome sequencing of a family with local anesthetic resistance. Minerva Anestesiol 2016;82:1089-97.
[57] Koval OM, Snyder JS, Wolf RM, Pavlovicz RE, Glynn P, Curran J, Leymaster ND, Dun W, Wright PJ, Cardona N, Qian L, Mitchell CC, Boyden PA, Binkley PF, Li C, Anderson ME, Mohler PJ, Hund TJ. Ca2+/calmodulin-dependent protein kinase II-based regulation of voltage-gated Na+ channel in cardiac disease. Circulation 2012;126:2084-94.

[58] Ortiz-Bonnin B, Rinne S, Moss R, Streit AK, Scharf M, Richter K, Stober A, Pfeufer A, Seemann G, Kaab S, Beckmann BM, Decher N. Electrophysiological characterization of a large set of novel variants in the SCN5A-gene: identification of novel LQTS3 and BrS mutations. Pflugers Arch 2016;468:1375-87.
[59] Paulussen A, Matthijs G, Gewillig M, Verhasselt P, Cohen N, Aerssens J. Mutation analysis in congenital Long QT Syndrome--a case with missense mutations in KCNQ1 and SCN5A. Genet Test 2003;7:57-61.
[60] Tester DJ, Valdivia C, Harris-Kerr C, Alders M, Salisbury BA, Wilde AA, Makielski JC, Ackerman MJ. Epidemiologic, molecular, and functional evidence suggest A572D-SCN5A should not be considered an independent LQT3-susceptibility mutation. Heart Rhythm 2010;7:912-9.
[61] Marjamaa A, Newton-Cheh C, Porthan K, Reunanen A, Lahermo P, Vaananen H, Jula A, Karanko H, Swan H, Toivonen L, Nieminen MS, Viitasalo M, Peltonen L, Oikarinen L, Palotie A, Kontula K, Salomaa V. Common candidate gene variants are associated with QT interval duration in the general population. J Intern Med 2009;265:448-58.
[62] Abbott GW, Sesti F, Splawski I, Buck ME, Lehmann MH, Timothy KW, Keating MT, Goldstein SA. MiRP1 forms IKr potassium channels with HERG and is associated with cardiac arrhythmia. Cell 1999;97:175-87.
[63] Gouas L, Nicaud V, Chaouch S, Berthet M, Forhan A, Tichet J, Tiret L, Balkau B, Guicheney P. Confirmation of associations between ion channel gene SNPs and QTc interval duration in healthy subjects. Eur J Hum Genet 2007;15:974-9.
[64] Priori SG, Napolitano C, Memmi M, Colombi B, Drago F, Gasparini M, DeSimone L, Coltorti F, Bloise R, Keegan R, Cruz Filho FE, Vignati G, Benatar A, DeLogu A. Clinical and molecular characterization of patients with catecholaminergic polymorphic ventricular tachycardia. Circulation 2002;106:69-74.
[65] Xiong HY, Alipanahi B, Lee LJ, Bretschneider H, Merico D, Yuen RK, Hua Y, Gueroussov S, Najafabadi HS, Hughes TR, Morris Q, Barash Y, Krainer AR, Jojic N, Scherer SW, Blencowe BJ, Frey BJ. RNA splicing. The human splicing code reveals new insights into the genetic determinants of disease. Science 2015;347:1254806.
[66] Anderson CL, Kuzmicki CE, Childs RR, Hintz CJ, Delisle BP, January CT. Large-scale mutational analysis of Kv11.1 reveals molecular insights into type 2 long QT syndrome. Nat Commun 2014;5:5535.
[67] Hedley PL, Jorgensen P, Schlamowitz S, Wangari R, Moolman-Smook J, Brink PA, Kanters JK, Corfield VA, Christiansen M. The genetic basis of long QT and short QT syndromes: a mutation update. Hum Mutat 2009;30:1486-511.
[68] Moss AJ, Shimizu W, Wilde AA, Towbin JA, Zareba W, Robinson JL, Qi M, Vincent GM, Ackerman MJ, Kaufman ES, Hofman N, Seth R, Kamakura S, Miyamoto Y, Goldenberg I, Andrews ML, McNitt S. Clinical aspects of type-1 long-QT syndrome by location, coding type, and biophysical function of mutations involving the KCNQ1 gene. Circulation 2007;115:2481-9.
[69] Riobello C, Gomez J, Gil-Pena H, Tranche S, Reguero JR, de la Hera JM, Delgado E, Calvo D, Moris C, Santos F, Coto-Segura P, Iglesias S, Alonso B, Alvarez V, Coto E. KCNQ1 gene variants in the risk for type 2 diabetes and impaired renal function in the Spanish Renastur cohort. Mol Cell Endocrinol 2016;427:86-91.
[70] Shamgar L, Ma L, Schmitt N, Haitin Y, Peretz A, Wiener R, Hirsch J, Pongs O, Attali B. Calmodulin is essential for cardiac IKS channel gating and assembly: impaired function in long-QT mutations. Circ Res 2006;98:1055-63.
[71] Zareba W, Moss AJ, Sheu G, Kaufman ES, Priori S, Vincent GM, Towbin JA, Benhorin J, Schwartz PJ, Napolitano C, Hall WJ, Keating MT, Qi M, Robinson J, Andrews ML, International Lqts Registry UoRRNY. Location of mutation in the KCNQ1 and phenotypic presentation of long QT syndrome. J Cardiovasc Electrophysiol 2003;14:1149-53.
[72] Baskar S, Ackerman MJ, Clements D, Mayuga KA, Aziz PF. Compound heterozygous mutations in the SCN5A-encoded Nav1.5 cardiac sodium channel resulting in atrial standstill and His-Purkinje system disease. J Pediatr 2014;165:1050-2.
[73] Benson DW, Wang DW, Dyment M, Knilans TK, Fish FA, Strieper MJ, Rhodes TH, George AL, Jr. Congenital sick sinus syndrome caused by recessive mutations in the cardiac sodium channel gene (SCN5A). J Clin Invest 2003;112:1019-28.

[74] Beyder A, Mazzone A, Strege PR, Tester DJ, Saito YA, Bernard CE, Enders FT, Ek WE, Schmidt PT, Dlugosz A, Lindberg G, Karling P, Ohlsson B, Gazouli M, Nardone G, Cuomo R, Usai-Satta P, Galeazzi F, Neri M, Portincasa P, Bellini M, Barbara G, Camilleri M, Locke GR, 3rd, Talley NJ, D'Amato M, Ackerman MJ, Farrugia G. Loss-of-function of the voltage-gated sodium channel NaV1.5 (channelopathies) in patients with irritable bowel syndrome. Gastroenterology 2014;146:1659-68.
[75] Butters TD, Aslanidi OV, Inada S, Boyett MR, Hancox JC, Lei M, Zhang H. Mechanistic links between Na+ channel (SCN5A) mutations and impaired cardiac pacemaking in sick sinus syndrome. Circ Res 2010;107:126-37.
[76] Celestino-Soper PB, Doytchinova A, Steiner HA, Uradu A, Lynnes TC, Groh WJ, Miller JM, Lin H, Gao H, Wang Z, Liu Y, Chen PS, Vatta M. Evaluation of the Genetic Basis of Familial Aggregation of Pacemaker Implantation by a Large Next Generation Sequencing Panel. PLoS One 2015;10:e0143588.
[77] Gui J, Wang T, Jones RP, Trump D, Zimmer T, Lei M. Multiple loss-of-function mechanisms contribute to SCN5A-related familial sick sinus syndrome. PLoS One 2010;5:e10985.
[78] Lawrence L, Sincan M, Markello T, Adams DR, Gill F, Godfrey R, Golas G, Groden C, Landis D, Nehrebecky M, Park G, Soldatos A, Tifft C, Toro C, Wahl C, Wolfe L, Gahl WA, Boerkoel CF. The implications of familial incidental findings from exome sequencing: the NIH Undiagnosed Diseases Program experience. Genet Med 2014;16:741-50.
